# Social media influencer or traditional celebrity? Moderational analysis on the relationship between endorser type and endorsement effectiveness

**DOI:** 10.1371/journal.pone.0326911

**Published:** 2025-07-01

**Authors:** Chen-Yueh Chen, Yi-Hsiu Lin, Tzu-Yun Yeh, Ya-Lun Chou, Tsu-Lin Yeh

**Affiliations:** 1 Doctoral Program for Transnational Sport Management and Innovation, National Taiwan Sport University, Taoyuan, Taiwan; 2 Master Program of Sport Facility and Health Promotion, National Taiwan University, Taipei, Taiwan; 3 Department of Health, Exercise Science, and Recreation Management, University of Mississippi, Oxford, Mississippi, United States of America; University of Missouri School of Medicine, UNITED STATES OF AMERICA

## Abstract

The effectiveness of an endorsement depends on the endorser. Social media influencers and traditional celebrities are both prized as endorsers by advertisers. Studies comparing the effectiveness of these two endorser types has yielded inconsistent results. The effectiveness of a type of endorser depends on the advertising context. The present study explored the moderating effects of product–endorser fit, popularity, self-congruity, similarity, and likeability on the relationship between endorser type and consumer attitudes toward an endorsement in two different sporting contexts. A multiple-factor, independent sample, pretest–posttest, quasi-experimental design was implemented. In total, 473 participants were enrolled in four experiments. Data were analyzed using two-way analysis of covariance and hierarchical linear regression. The results indicate that product–endorser fit, popularity, self-congruity, and similarity but not likeability moderate the relationship between endorser type and consumer attitudes toward an endorsement. This study contributes to the literature on endorsement marketing and helps marketers more effectively choose an endorser that fits the given marketing context.

## Introduction

Endorsements from social media influencers (SMIs) are popular given the ubiquity of social media [1] and have displaced endorsements from traditional celebrities [2]. SMIs gain popularity by branding themselves as experts. Traditional celebrities become famous by performing on screen or making music [3]. Most individuals are likely to have seen an SMI at least once. Endorsements from traditional celebrities are no longer the only endorsements that are highly prized by advertisers [1,4]. Empirical findings have demonstrated the effectiveness of traditional celebrity endorsements in advertising. The growing prominence of SMIs has created a need for research into the distinct marketing effects of SMI endorsements. The effectiveness and psychological mechanisms behind SMI endorsements are important areas of research.

The effectiveness of different endorser types (i.e., SMIs and traditional celebrities) depends on the specific context. Endorsements from traditional celebrities have been demonstrated to be effective [5–8]. Endorsements from noncelebrities have also been demonstrated to be effective [9–12]. An empirical study demonstrated that traditional celebrities are more persuasive than are SMIs when endorsing luxury brands and brands targeting older consumers and that SMIs are more persuasive than are traditional celebrities when endorsing brands that target younger consumers [13]. By contrast, other studies have argued that SMIs and traditional celebrities are equally effective endorsers [14,15]. These inconsistent findings in the literature suggest that different types of endorsers are effective in different contexts.

A meta-analysis of empirical studies investigated the factors that affect the relationship between endorser type and endorsement effectiveness [15]. Product–endorser fit has been comprehensively examined in marketing research [16]. The relationship between self-congruity and endorser has also been examined [17]. Popularity [18], similarity [16], and likeability [16] are factors that affect endorsement effectiveness. Scholars have called for greater innovation in endorser marketing to respond to the advancements in digital technologies that have occurred and the rise of SMIs, who have changed how traditional celebrity endorsements are perceived [1]. Accordingly, investigating the factors that affect the relationship between endorser type and endorsement effectiveness is essential.

The size of the sports market has been increasing. The forces driving this growth include the growing popularity of organized sporting events [19]. Empirical research into the effectiveness of endorsements in sporting advertising, particularly digital advertising, remains limited [20,21]. Advancements in social media marketing and the sports industry warrant renewed attention from researchers, who should investigate novel, digital avenues for endorsements in advertising. Studies investigating endorsement effectiveness should involve multiple contexts and diverse regions [1]. The present study was conducted in Asia as a response to the call for further research in this region.

Advancements in technology and social media have prompted the need to re-examine how endorsements function in marketing and how SMIs and traditional celebrities affect endorsements. Researchers are encouraged to explore this issue from more diverse perspectives. Studies have argued that research into SMI endorsements should incorporate moderating factors when establishing theories and testing relationships [22]. Accordingly, the present study examined the moderating effects of product–endorser fit, popularity, self-congruity, similarity, and likeability on the relationship between endorser type (SMI vs. traditional celebrity) and endorsement effectiveness in two sporting scenarios (a spectator sporting event and sporting goods).

## Literature review

### Theoretical background

**Fig 1:**
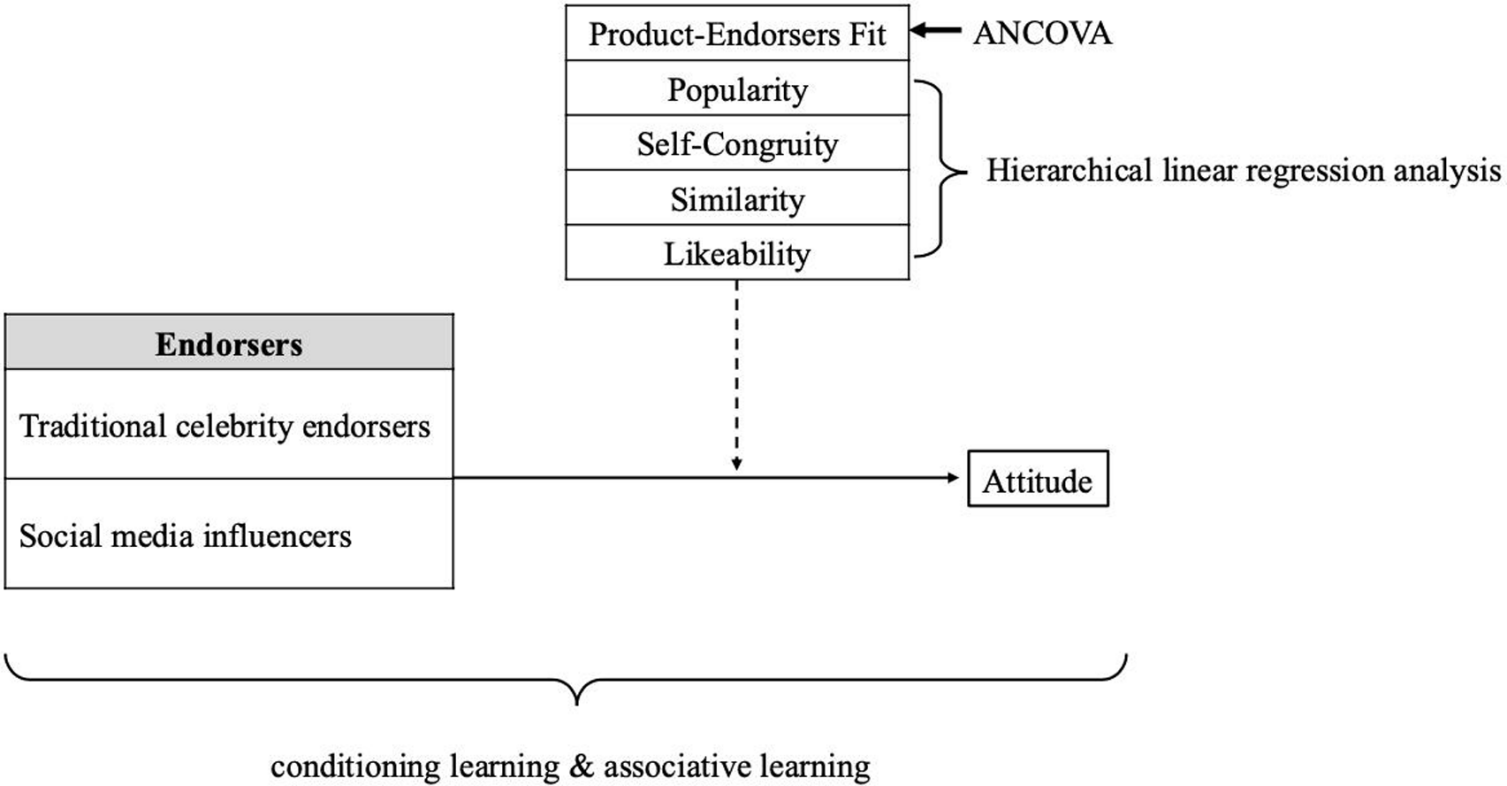
Research framework. Note: Dashed lines indicate moderating effects.

Multiple theoretical frameworks have been employed in endorsement marketing research [1]. This study adopted the two concepts of conditioning and associative learning [23]. The primary function of conditioning is to establish an association between a conditioned stimulus and an unconditioned stimulus, with this association eventually eliciting a conditioned response to the unconditioned stimulus. In the context of sporting events, the repeated pairing of an endorser with an event strengthens the association between the two, leading to a conditioning effect [24]. The application of conditioning theory in endorser research has been questioned by some scholars [25] and supported by others [26,27]. Similar to conditioning theory, associative learning theory suggests that the connection between two concepts or objects is influenced by an individual’s experience or perceived associations and that this connection can be reinforced through repeated pairings [28–30]. In associative learning, “learning can consistently occur when links are made to connect pieces of seemingly unconnected information such that when one piece of information is called to mind, it automatically triggers the other” [31,32]. When a link between two seemingly unconnected pieces of information is formed, it dwells in the same association set within one’s memory. To illustrate, a person who thinks about having an ice cream may simultaneously think of summer afternoons at a baseball game [31]. Ice cream and baseball games are two seemingly unconnected pieces of information. Nevertheless, a linkage is created because ice cream and baseball games inhabit the same association set. Studies have demonstrated that associative learning mechanisms can influence consumer attitudes [33,34] and can operate subconsciously [35,36]. The present study considered spectator sporting events and sporting goods as unconditioned stimuli and SMI and traditional celebrity endorsers as conditioned stimuli. The present study assessed participants’ attitudes toward these stimuli (Fig 1).

### Endorsements in sporting contexts

Research on endorsements in sporting contexts has demonstrated that endorsers influence consumer attitudes toward advertisements [24,37–41] and perceptions of brands [42]. When a negative incident occurs, an endorser’s influence often extends beyond the brand they represent, shaping perceptions of other similar brands [43]. Athlete endorsements have been a major focus in sports marketing research [37–39]. By contrast, SMI endorsements, which have a distinct role in sports marketing, have received little attention in this research field. Given that sports fans strongly identify with the sports teams that they support, investigating the factors that affect the relationship between endorser type and endorsement effectiveness can provide valuable insights. Fan identity and team loyalty are factors affecting the ability of SMIs to shape how loyal fans are to a team [44]. An investigation into how SMIs influence sports fans specifically is essential given that the influence of an SMI depends on context and that fan identity and loyalty are distinct concepts in sports marketing [45,46].

### Social media influencers

Several studies have explored the marketing power and advertising effectiveness of SMIs (Table 2) [47,48]. Most studies on SMIs have adopted quantitative, empirical research designs, focusing on factors such as self-concept [49], similarity [50], paid endorsements [51], and likability [52]. One study (a systematic review) from 2014 to 2023 investigated the evolving trends associated with traditional celebrity and SMI endorsements [1]. Another study investigated how SMI follower count affects purchase intention toward the endorsed brand [53].

**Table 1:** Summary of participant demographic characteristics.

Variable		Experiment 1		Experiment 2		Experiment 3		Experiment 4	
n	%	n	%	n	%	n	%
Sex	Male	62	50.8%	47	38.5%	51	50.5%	44	34.4%
	Female	60	49.2%	75	61.5%	50	49.5%	84	65.6%
Age	20-30	105	86.1%	116	95.1%	27	26.7%	38	29.7%
	31-40	15	12.3%	2	1.6%	55	54.5%	62	48.4%
	41-50	2	1.6%	3	2.5%	2	2.0%	4	3.1%
	51-60	0	0%	1	0.8%	10	9.9%	8	6.3%
	61&above	0	0%	0	0%	7	6.9%	16	12.5%
Education	H/S & below	14	11.5%	3	2.5%	4	4.0%	11	8.6%
	Bachelor	81	66.4%	114	93.4%	59	58.4%	79	61.7%
	Master/Doctor	27	22.1%	5	4.1%	38	37.6%	38	29.7%

**Table 2:** Summary of hierarchical regression analysis for experiment 1.

		pre AD	SMI	Decentered	SMI^*^ Decentered	R^2^	ΔR^2^	Δ*F*
popularity	Model 1	0.469*				0.220	0.220	33.924*
	Model 2	0.412*	−0.130	0.375^*^		0.381	0.160	15.274*
	Model 3	0.404*	−0.130	0.470^*^	−0.133	0.390	0.009	1.691
self- congruity	Model 1	0.469*				0.220	0.220	33.924*
	Model 2	0.258*	0.030	0.608*		0.536	0.315	40.079*
	Model 3	0.259*	0.030	0.577*	0.043	0.537	0.001	0.235
similarity	Model 1	0.469*				0.220	0.220	33.924*
	Model 2	0.305*	0.010	0.547*		0.490	0.269	31.104*
	Model 3	0.305*	0.010	0.501*	0.066	0.492	0.002	0.502
likeability	Model 1	0.469*				0.220	0.220	33.924*
	Model 2	0.361*	−0.057	0.439*		0.415	0.195	19.636*
	Model 3	0.362*	−0.056	0.474*	−0.048	0.416	0.001	0.208

Note:

* indicates *p* < .05; ΔR² represents change in R²; ΔF represents change in F.

An empirical study explored how SMIs affect followers in the equestrian market [54]. Other studies have explored how college athletes serving as SMIs affect consumer perceptions [55,56]. More research on SMIs in sports marketing is necessary. Building on the empirical findings and theoretical frameworks in the literature, the present study investigated the effectiveness of SMI endorsements in sporting contexts, with a focus on underexplored moderating variables.

### Hypotheses on moderating effects

#### Product–endorser fit.

Product–endorser fit refers to the degree in which the image, personality, or expertise of an endorser fits the advertised product [16]. Numerous studies have indicated that the level of fit between an endorser and the endorsed brand is positively associated with endorsement effectiveness [17,41,57–59]. SMI endorsements are more effective than are traditional celebrity endorsements [50]. Instagram users exhibit more favorable attitudes toward brands endorsed by SMIs than toward brands endorsed by traditional celebrities [12,60]. Some studies have demonstrated that in cases of low product–endorser fit, SMI endorsements are likely to outperform traditional celebrity endorsements because SMI endorsements tend to involve greater social media interactivity and platform penetration, which lead to enhanced brand attitudes [61,62]. Other studies have demonstrated that traditional celebrities outperform SMIs regardless of the level of product–endorser fit [63]. The inconsistency between these empirical findings led us to pose Research Question (RQ) 1.

RQ 1: Does product–endorser fit moderate the relationship between endorser type and consumer attitudes toward advertisement?

#### Popularity.

The popularity of an SMI is measured in terms of the preferences of their followers, their number of followers, and the numbers of comments on their posts [64]. SMI popularity affects the number of likes [65] and influences consumer purchasing behavior [66,67]. SMI follower count is positively correlated with SMI attractiveness, which in turn increases the willingness of followers to purchase endorsed products [68]. In endorsement research, perceived endorser popularity has been examined as an independent variable and as a mediator [69]; perceived endorser popularity may also be a moderator. To illustrate, the effect of SMI popularity on perceived authenticity is more prominent among those who believe that SMIs are generally self-serving when endorsing products [18]. RQ 2 was thus formulated as follows.

RQ 2: Does popularity moderate the relationship between endorser type and consumer attitudes toward advertisement?

#### Self-congruity.

Self-congruity refers to “the likeliness of comparing oneself with other objects and stimuli” [70]. Self-congruity is regarded as an extension of the notion of self-concept. Consumers prefer products or services that reflect their self-concept and image [71]. Sirgy [72] proposed that self-congruity has four dimensions that correspond to an individual’s actual, ideal, social, and ideal social selves. Consumers may use endorsed products to align themselves with the values associated with the endorsed brand and establish and maintain their sense of self-congruity [73]. Social influence theory suggests that an individual’s attitudes and behaviors are influenced by others, with the level of influence depending on how the individual perceives the source of influence [74]. Under this theory, endorsers can be regarded as reference groups that affect consumer attitudes and behaviors. Higher levels of self-congruity between a consumer and an endorser have been associated with increased endorsement effectiveness [17,49] and repurchase intention [38,71]. Endorser–consumer congruency is more important in SMI endorsements than in traditional celebrity endorsements [75]. RQ 3 was thus formulated as follows.

RQ 3: Does self-congruity moderate the relationship between endorser type and consumer attitudes toward advertisement?

#### Similarity.

In endorsement advertising, similarity refers to the extent to which an individual identifies with an endorser [76]. Specifically, similarity is depicted as a perceived resemblance between the endorser and the receiver of the message [77]. Individuals who perceive a higher level of similarity between themselves and a celebrity endorser tend to exhibit more favorable attitudes toward a given advertisement and brand and exhibit stronger purchase intentions [78–80]. Consumers perceive endorsers to be key reference groups, which are groups that influence consumer evaluations and behaviors [81,82]. According to Evan Varsamis [83], brands selecting SMI endorsers should consider the similarity between the target audience and the SMI because perceived similarity plays a crucial role in the relationship between consumers and endorsers. Individuals tend to perceive greater similarity with SMIs than with celebrities. Accordingly, SMI endorsers may have more value than traditional celebrity endorsers [16]. RQ 4 was thus formulated as follows.

RQ 4: Does similarity moderate the relationship between endorser type and consumer attitudes toward advertisement?

#### Likability.

Likeability indicates the degree to which an endorser is perceived to be cooperative, friendly, minimally aggressive, and highly prosocial [84]. Likability involves an individual’s emotional response to another’s physical traits, behavior, or other characteristics [85]. Research on social psychology has indicated that likability is correlated with persuasiveness [86], that high likability in advertising improves brand attitudes [87], and that more likable SMIs are more persuasive [78,82]. Brands endorsed by well-liked SMIs tend to benefit from more favorable brand attitudes and higher purchase intentions [88]. Having a larger follower base enhances the likability of an SMI, thereby increasing their influence [69]. Likeability significantly predicts parasocial interaction in social media scenarios [89]. In the context of the entertainment industry, likeability toward a celebrity is found to be most appealing to the audience [90]. Likeability may moderate the relationship between endorser type and endorsement effectiveness. RQ 5 was thus formulated as follows.

RQ 5: Does likability moderate the relationship between endorser type and consumer attitudes toward advertisement?

## Methods

### Research settings

To enhance its external validity, this study examined the effectiveness of SMI endorsements in two sporting contexts, specifically a spectator sporting event (a badminton tournament) and sporting goods (basketball shoes). Taiwanese badminton players excel internationally, and the Taiwan Professional Basketball League and Plus League aim to be Taiwan’s premier professional basketball leagues. These leagues organize popular events and provide a key platform for Taiwanese basketball talent [91]. Badminton and basketball are two of the most popular sports in Taiwan.

### Research design and stimuli

To mitigate the influence of endorser sex on internal validity, this study was conducted using scenarios with either all-male or all-female endorsers. Endorsement of a spectator sporting event and endorsement of a sporting goods were the two contexts used as a basis for the following four experiments:

**Experiment 1** Male SMI; male traditional celebrity; sporting goods scenario.

**Experiment 2** Female SMI; female traditional celebrity; sporting goods scenario.

**Experiment 3:** Male SMI; male traditional celebrity; spectator sporting event scenario.

**Experiment 4:** Female SMI; male traditional celebrity; spectator sporting event scenario.

For Experiment 1, an independent sample, pretest–posttest, between-subjects design was adopted, and participants were randomly assigned to one of four groups in a 2 (type of endorser: male SMI vs. male traditional celebrity) × 2 (level of product–endorser fit: high vs. low) design. The sporting good that was endorsed was basketball shoes. Experiment 2 was identical to Experiment 1 except that the SMI and traditional celebrity were female instead of male. Experiment 3 was identical to Experiment 1 except that a sporting event served as the research object instead of basketball shoes. Finally, Experiment 4 was nearly identical to Experiment 2 except that a sporting event served as the research object instead of basketball shoes.

The SMIs and traditional celebrities used in this research were selected by 30 research participants, who voted for them. Regarding the level of product–endorser fit, the outcome for voting endorsers generated high-fit versus low-fit endorsers. The SMIs and traditional celebrities that were selected by the participants were incorporated into research posters that served as the experimental stimuli in this research. In line with the experimental stimuli used in [92], in which sports apparel was displayed with a celebrity in a photo, the selected endorsers were incorporated into the experimental posters and juxtaposed with a sporting event logo.

### Participants and research procedure

Table 1 provides a summary of the demographic characteristics of the participants in each experiment. Per criteria developed by Noordzij, Tripepi [93], a sample size of 120 for the present study was deemed sufficient for a significance level of 0.05 and a power level of 0.8. All of the experiments except for Experiment 3 met this sample size criterion; therefore, the sample sizes for the experiments in this research were considered to be acceptable.

This study was approved by the Research Ethics Office of National Taiwan University (protocol code 202012ES034, approval date 20210611). The recruited participants were briefed on the study and asked to provide their written consent. Data were collected in February and March 2022 using an online survey. An online survey was considered appropriate given the predominantly online nature of social media marketing. For the spectator sporting event context, participants were recruited from online fan communities for the Yonex Taipei Open, a badminton tournament. For the sporting goods scenario, participants were recruited from online road racing communities. Recruitment information and a link to the survey was posted on social media platforms. Individuals aged 20 years or older were eligible to participate. In the spectator sporting event scenario, participants were randomly assigned to either an SMI endorsement group or a traditional celebrity endorsement group. Convenience sampling was performed in this research due to the difficulty of implementing random sampling in this context; thus, the study design is quasi-experimental.

The recruited participants were briefed on the study by reading the description regarding this research and asked to provide consent by signing an electronic consent form. The participants then completed the online survey, which started with a pretest measuring their brand attitude regarding the Yonex Taipei Open. Subsequently, the participants were exposed to the experimental treatment (see appendix) and then asked to complete post-test brand attitude measures. The participants in the SMI endorsement group viewed a poster featuring an SMI endorsement, whereas those in the traditional celebrity endorsement group viewed a poster with a traditional celebrity endorsement. Each group was exposed to their respective poster five times for 15 seconds, without breaks between each repetition. This followed an established experimental protocol [94] designed to form a linkage by using conditioning and associative learning. The participants further responded to questions related to the moderator variables, namely, popularity, self-congruity, similarity, and likability. Finally, the participants provided information on their demographic characteristics. The research process was consistent across the spectator sporting event and sporting goods scenarios, with the only variation being the nature of the endorsement scenario.

### Manipulation check

Manipulation checks on the level of product–endorser fit were performed to ensure accuracy [32] in accordance with a scale developed by Till and Busler [32]. Four 7-point semantic differential measurement items were used: “Compatible/Not Compatible,” “Good Fit/Bad Fit,” “Relevant/Irrelevant,” and “Good Match/Bad Match.” Independent samples *t*-tests revealed that the scores for the manipulation check items were significantly higher in the high-fit group than in the low-fit group for Experiment 1 (M_High-Fit _= 4.59; SD_High-Fit _= 1.90; M_Low-Fit _= 2.93; SD_Low__-Fit_ = 1.87; *t* = 4.86, *p* < .01), Experiment 3 (M_High-Fit_ = 5.03; SD_High-Fit_ = 1.91; M_Low-Fit_ = 2.95; SD_Low-Fit_ = 2.14; *t* = 5.18, *p* < .01), and Experiment 4 (M_High-Fit_ = 4.55; SD_High-Fit_ = 1.80; M_Low-Fit_ = 3.73; SD_Low-Fit_ = 1.91; *t* = 5.32, *p* < .01), indicating effective manipulations. The manipulation check was not effective for Experiment 2 (M_High-Fit_ = 3.62; SD_High-Fit_ = 2.10; M_Low-Fit_ = 2.31; SD_High-Fit_ = 2.04; *t* = 0.84, *p* = 0.401).

### Research instruments

The dependent variable in this research was attitudes toward advertisement, which coincides with the empirical findings proposed by Hariningsih and others [1]. The independent variable was endorser type, which was manipulated across two different sporting contexts. The present study further explored the moderating effects of product–endorser fit, popularity, self-congruity, similarity, and likability on the relationship between endorser type and consumer attitudes toward the advertisement. The following variables were measured: attitudes toward advertisement, popularity, self-congruity, similarity, and likability.

#### Attitudes toward advertisement.

The scale for measuring attitudes toward advertisement was adapted from Choi and Rifon (17) and comprised five semantic differential items measured on a 7-point scale: “Good/Bad,” “Favorable/Unfavorable,” “Unpleasant/Pleasant,” “Boring/Interesting,” and “Like/Dislike.” According to Hair, Black [95], a measurement instrument is considered to be valid and reliable if its Cronbach’s alpha and average variance extracted (AVE) are above 0.8 and 0.5, respectively. The Cronbach’s alpha for this scale ranged from 0.92 to 0.98, indicating satisfactory internal consistency. The AVE values ranged from 0.72 to 0.85, suggesting satisfactory convergent validity.

#### Popularity.

The scale used to measure popularity was adapted from Ladhari, Massa and Skandrani (67) and comprised the following four items: “This endorser is quite famous,” “This endorser has a large number of followers,” “This endorser’s popularity is rapidly increasing,” and “This endorser’s posts attract a high level of engagement.” Items were rated on a 7-point Likert-type scale with endpoints ranging from 1 (*strongly disagree*) to 7 (*strongly agree*). The Cronbach’s alpha for this scale ranged from 0.90 to 0.97, indicating satisfactory internal consistency. The AVE values ranged from 0.70 to 0.90, indicating satisfactory convergent validity.

#### Self-congruity.

The scale for assessing self-congruity was adapted from Usakli and Baloglu [96] and measured actual and ideal self-congruity. Actual self-congruity was assessed using three items: “This endorser is consistent with how I view myself,” “I feel that I have similar characteristics as this endorser,” and “This endorser’s traits align with my self-perception.” Ideal self-congruity was measured using three items: “This endorser aligns with how I wish to see myself,” “I want to share traits with this endorser,” and “This endorser’s traits are consistent with my ideal self-image.” Items were rated on a 7-point Likert-type scale with endpoints ranging from 1 (*strongly disagree*) to 7 (*strongly agree*). The Cronbach’s alpha for this scale ranged from 0.95 to 0.98, indicating satisfactory internal consistency. The AVE values ranged from 0.77 to 0.93, indicating satisfactory convergent validity.

#### Similarity.

The scale for assessing similarity was adapted from Taillon, Mueller (82) and comprised three items: “This endorser is similar to me,” “I feel that I am similar to this endorser,” and “Overall, I believe that I am similar to this endorser.” Items were rated on a 7-point Likert-type scale with endpoints ranging from 1 (*strongly disagree*) to 7 (*strongly agree*). The Cronbach’s alpha for this scale ranged from 0.97 to 0.99, indicating satisfactory internal consistency. The AVE values ranged from 0.91 to 0.98, indicating satisfactory convergent validity.

#### Likeability.

The scale for measuring likeability was adapted from Whittler and DiMeo [97] and comprised four semantic differential items measured on a 7-point scale: “Cold/Warm,” “Likable/Dislikable,” “Sincere/Insincere,” and “Friendly/Unfriendly.” The Cronbach’s alpha for this scale ranged from 0.95 to 0.98, indicating satisfactory internal consistency. The AVE values ranged from 0.77 to 0.95, indicating satisfactory convergent validity.

### Data analysis

Two-way analysis of covariance (ANCOVA) was used to test the moderating effect of product–endorser fit on the relationship between endorser type and endorsement effectiveness. In the ANCOVA model, pretest scores served as the covariate and product–endorser fit and endorser type were independent variables. The significant interaction term of the independent variables indicated a moderating effect. Hierarchical linear regression analysis was conducted to examine the moderating effects of popularity, self-congruity, similarity, and likeability. Pretest outcomes formed the first block, endorser type (formulated as a dummy variable) and the moderating variable formed the second block, and the interaction term for endorser type and the moderating variable formed the third block. The significant interaction term between endorser type and the moderating variable was taken to suggest a moderating effect.

## Results

### Experiment 1: male endorsers/sporting goods

Attitudes toward advertisement was significant in the ANCOVA model (F = 17.313, *p* < .01, R^2^ = 0.372). The interaction between endorser type and product–endorser fit (high vs. low) was significant (F = 4.05, *p* = .046, η^2^ = 0.033). A further analysis of the interaction effect indicated that when the level of product–endorser fit was high, the traditional celebrity endorsement group exhibited more positive attitudes toward advertisement relative to the SMI endorsement group (M_Social Media Influencer_ = 4.35, SE_Social Media Influencer_ = 0.22, M_Traditional Celebrity_ = 5.31, SE_Traditional Celebrity_ = 0.23, F = 8.827, *p* = .004, η^2^ = 0.132). No significant difference in attitudes toward advertisement was observed between the SMI endorsement group and the traditional celebrity endorsement group when product–endorser fit was low (M_Social Media Influencer_ = 3.69, SE_Social Media Influencer_ = 0.26, M_Traditional Celebrity_ = 3.66, SE_Traditional Celebrity_ = 0.26, F = 0.006, *p* = .940, η^2^ = 0.000).

**Fig 2:**
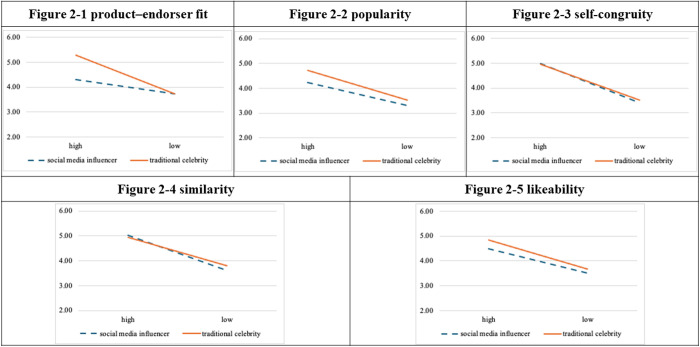
Moderational diagrams for experiment 1.

Hierarchical linear regression analysis (Table 2. and Fig 2.) revealed that popularity (ΔF = 1.691, *p* = 0.196), self-congruity (ΔF = 0.235, *p* = 0.629), similarity (ΔF = 0.502, *p* = 0.480), and likeability (ΔF = 0.208, *p* = 0.649) did not moderate the relationship between endorser type and attitudes toward advertisement.

### Experiment 2: Female endorsers/sporting goods

Due to the ineffectiveness of the manipulation on product–endorser fit, no further analysis on interaction between endorser type and product–endorser fit (high vs. low) was performed.

**Fig 3:**
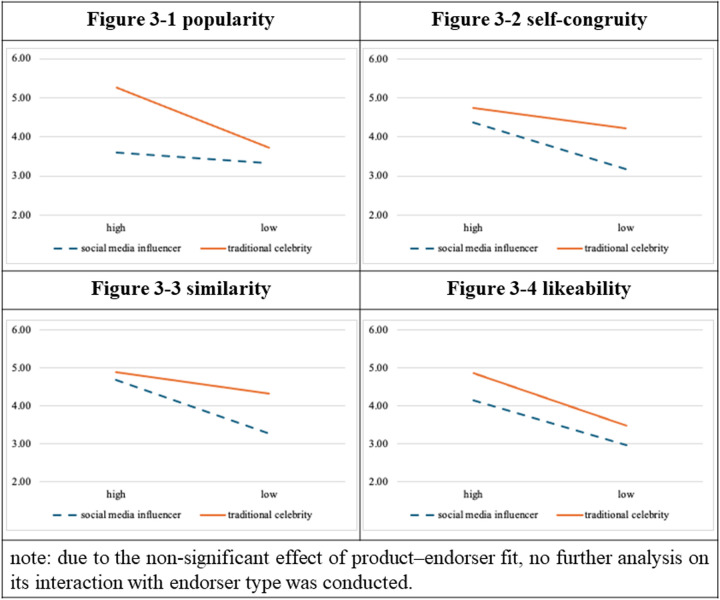
Moderational diagrams for experiment 2.

**Fig 4:**
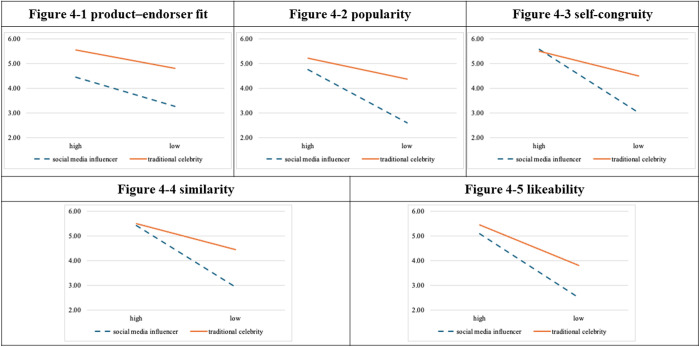
Moderational diagrams for experiment 3.

The hierarchical linear regression analysis. (Table 3. and Fig 3.) indicated that the interaction between endorser type and popularity was significant (ΔR² = 0.034, ΔF = 7.000, *p* = 0.009, Fig 4). That is, popularity moderated the relationship between endorser type and attitudes toward advertisement. Self-congruity (ΔF = 0.710, *p* = 0.401), similarity (ΔF = 2.152, *p* = 0.145), and likability (ΔF = 0.539, *p* = 0.464) did not moderate the relationship between endorser type and attitudes toward advertisement.

**Table 3:** Summary of hierarchical regression analysis for Experiment 2.

		pre AD	SMI	Decentered	SMI^*^ Decentered	R^2^	ΔR^2^	Δ*F*
popularity	Model 1	0.418^*^				0.175	0.175	25.383*
	Model 2	0.288^*^	−0.327*	0.375*		0.396	0.221	21.605*
	Model 3	0.267^*^	−0.332*	0.602	−0.290*	0.430	0.034	7.000*
self- congruity	Model 1	0.418^*^				0.175	0.175	25.383*
	Model 2	0.259^*^	−0.210*	0.370*		0.376	0.201	19.007*
	Model 3	0.260^*^	−0.211*	0.303*	0.091	0.379	0.004	0.710
similarity	Model 1	0.418^*^				0.175	0.175	25.383*
	Model 2	0.315^*^	−0.244*	0.263*		0.325	0.150	13.111*
	Model 3	0.317^*^	−0.248*	0.134	0.169	0.337	0.012	2.152
likeability	Model 1	0.418^*^				0.175	0.175	25.383*
	Model 2	0.354^*^	−0.181*	0.471*		0.471	0.296	32.987*
	Model 3	0.354^*^	−0.181*	0.543*	−0.086	0.473	0.002	0.539

Note:

* indicates *p* < .05; ΔR² represents change in R²; ΔF represents change in F.

### Experiment 3: Male endorsers/sporting event

The ANCOVA results revealed that product–endorser fit (F = 0.487, p = 0.487, η^2^ = 0.005) did not moderate the relationship between endorser type and attitudes toward advertisement.

The hierarchical linear regression analysis (Table 4. and Fig 4.) demonstrated that the interaction between endorser type and self-congruity was significant (ΔR² = 0.049, ΔF = 8.937, *p* = 0.004). That is, self-congruity moderated the relationship between endorser type and attitudes toward advertisement. The interaction between endorser type and similarity was also significant (ΔR² = 0.049, ΔF = 8.880, *p* = 0.004). That is, similarity moderated the relationship between endorser type and attitudes toward advertisement. Popularity (ΔF = 0.299, *p* = 0.586) and likability (ΔF = 1.467, *p* = 0.229) did not moderate the relationship between endorser type and attitudes toward advertisement.

**Table 4:** Summary of hierarchical regression analysis for Experiment 3.

		pre AD	SMI	Decentered	SMI^*^ Decentered	R^2^	ΔR^2^	Δ*F*
popularity	Model 1	0.343*				0.117	0.117	13.161*
	Model 2	0.203*	−0.145	0.501*		0.425	0.308	25.945*
	Model 3	0.203*	−0.150	0.434*	0.078	0.427	0.002	0.299
self- congruity	Model 1	0.343*				0.117	0.117	13.161*
	Model 2	0.089	−0.149	0.540*		0.423	0.306	25.747*
	Model 3	0.126	−0.143	0.286*	0.327^*^	0.473	0.049	8.937*
similarity	Model 1	0.343*				0.117	0.117	13.161*
	Model 2	0.086	−0.169^*^	0.530*		0.419	0.301	25.137*
	Model 3	0.140	−0.165^*^	0.266*	0.331^*^	0.468	0.049	8.880*
likeability	Model 1	0.343*				0.117	0.117	13.161*
	Model 2	0.098	−0.074	0.666*		0.546	0.429	45.827*
	Model 3	0.099	−0.081	0.546*	0.144	0.553	0.007	1.467

Note:

* indicates *p* < .05; ΔR² represents change in R²; ΔF represents change in F.

### Experiment 4: Female endorsers/sporting event

The ANCOVA results revealed that product–endorser fit (F = 1.38, *p* = 0.243, η^2^ = 0.011) did not moderate the relationship between endorser type and attitudes toward advertisement.

**Fig 5:**
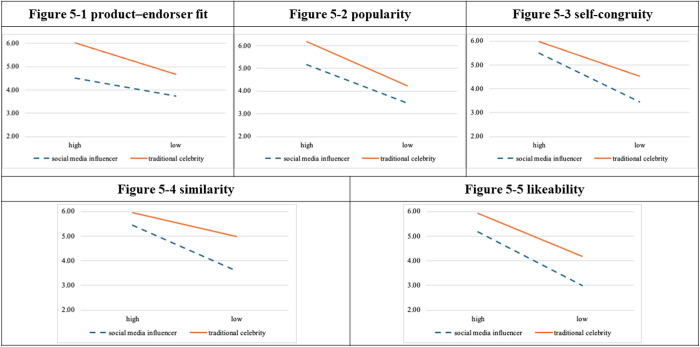
Moderational diagrams for experiment 4.

The hierarchical linear regression analysis (Table 5. and Fig 5.) revealed that popularity (ΔF = 1.080, *p* = 0.301) and likability (ΔF = 3.059, *p* = 0.083) did not moderate the relationship between endorser type and attitudes toward advertisement. Nevertheless, the interaction between endorser type and self-congruity was significant (ΔR² = 0.032, ΔF = 9.562, *p* = 0.002), meaning that self-congruity moderated the relationship between endorser type and attitudes toward advertisement. The interaction between endorser type and similarity was significant (ΔR² = 0.037, ΔF = 9.625, *p* = 0.002), demonstrating that similarity moderated the relationship between endorser type and attitudes toward advertisement.

**Table 5:** Summary of hierarchical regression analysis for experiment 4.

		pre AD	SMI	Decentered	SMI* Decentered	R^2^	ΔR^2^	ΔF
popularity	Model 1	0.431*				0.186	0.186	28.767*
	Model 2	0.175*	−0.167*	0.610*		0.602	0.416	64.904*
	Model 3	0.174*	−0.168*	0.678*	−0.090	0.606	0.003	1.080
self- congruity	Model 1	0.413*				0.186	0.186	28.767*
	Model 2	0.115	−0.133	0.614*		0.556	0.370	51.759*
	Model 3	0.123*	−0.097	0.457*	0.248^*^	0.588	0.032	9.562*
similarity	Model 1	0.431*				0.186	0.186	28.767*
	Model 2	0.157*	−0.218*	0.495*		0.490	0.304	36.976*
	Model 3	0.157*	−0.180*	0.347*	0.255^*^	0.527	0.037	9.625*
likeability	Model 1	0.431*				0.186	0.186	28.767*
	Model 2	0.178*	−0.164*	0.651*		0.655	0.469	84.462*
	Model 3	0.186*	−0.164*	0.541*	0.142	0.664	0.008	3.059

Note:

* indicates p < .05; ΔR² represents change in R²; ΔF represents change in F.

The findings from the four experiments indicate the following. Product–endorser fit and popularity moderate the relationship between the type of endorser and consumer attitudes toward an endorsement. Greater scores in attitude were obtained by the high-fit group than by the low-fit group. Similarly, scores for attitudes toward advertisement were higher in the high-popularity group than in the low-popularity group. Self-congruity and similarity moderated the relationship between endorser type and attitudes toward advertisement, and the endorsement tended to be more effective in the low-level group than in the high-level group. Likeability did not moderate the relationship between endorser type and endorsement effectiveness.

## Discussion

### General discussion

Informed by theories of conditioning and associative learning, this study compared the effectiveness of SMI endorsements with that of traditional celebrity endorsements in the field of sports marketing. Conditioning and associative learning describe how an individual forms an automatic conditioned response when exposed to a specific stimulus by linking pieces of seemingly unconnected information. Correspondingly, this study explored whether several specific variables moderate the relationship between endorser type and endorsement effectiveness by linking the type of endorser (SMIs and traditional celebrities) with different research objects (a spectator sporting event and sporting goods). The findings from the four experiments conducted in this study demonstrate that product–endorser fit, popularity, self-congruity, and similarity but not likeability moderate the relationship between endorser type and consumer attitudes toward advertisement. The theoretical implications of these findings are as follows.

First, traditional celebrity endorsers outperformed SMIs in scenarios involving high product–endorser fit, suggesting that consumers still prioritize traditional celebrities over SMIs in the tested scenarios [66,67]. Evidently, sports consumers still place considerable value on traditional celebrities. This finding is consistent with those of studies emphasizing that consumers exhibit more favorable brand attitudes toward brands endorsed by Instagram SMIs than toward those endorsed by traditional celebrities [12,60]. This finding underscores a sustained preference for well-established figures who align with the products they endorse, affirming the continued effect of traditional endorsements in sporting contexts [68].

Second, the present study identified endorser popularity as a key moderating factor. Traditional celebrity endorsers who are more popular have a stronger positive influence on consumer attitudes. This finding is consistent with those of other studies [17,59]. SMIs benefit from direct digital engagement. SMIs have less influence than traditional celebrities, who often possess broader visibility and reach. In high-visibility contexts, such as sports marketing, having sufficient reach is essential. Traditional celebrities have established mainstream presences and widespread media exposure, which amplify consumer attitudes. By contrast, SMIs do not have the same reach.

Third, self-congruity and similarity significantly moderated the relationship between endorser type and endorsement effectiveness. Under conditions of low self-congruity, the traditional celebrity endorsements were more effective than were the SMI endorsements. This finding contradicts those of other studies that have demonstrated that endorser–consumer congruency engenders more positive benefits to SMI endorsers than to traditional celebrity endorsers [75]. Similarly, traditional celebrity endorsements appear to be more effective than SMI endorsements under conditions of low similarity. Individuals perceive themselves as more similar to influencers than to celebrities [16]. When endorser–consumer congruity and similarity is low, consumers tend to respond more favorably to celebrities than to SMIs.

Likeability did not have a moderating effect on the relationship between endorser type and attitudes toward advertisement. Likeability toward a celebrity is found to be most appealing to the audience in the context of entertainment celebrity [90]. The effect of endorser likeability on endorsement effectiveness does not differ between traditional celebrities and SMIs.

Fourth, the present study contributes to the marketing literature by revealing nuanced differences in the effectiveness of SMIs and traditional celebrities in the area of sports marketing. Other studies have emphasized the authenticity and niche appeal of SMIs as key drivers of consumer engagement [50,71,81,98]. Our findings suggest that traditional celebrities outperform SMIs under conditions of high product–endorser fit, high popularity, low self-congruity, and low similarity, highlighting that consumers still prioritize traditional celebrities over SMIs in certain circumstances.

Finally, the present study highlights a need for further research to examine how other product categories and consumer segments engage with SMI endorsers. SMIs have gained prominence in digital marketing; nevertheless, SMI endorsements have limited effectiveness, particularly when compared with traditional celebrity endorsements. Sports marketers must carefully select endorsers to ensure effective alignment with consumer preferences. SMI endorsers are not universally more effective than are traditional celebrity endorsers. Consumer–brand relationships in sports marketing are highly nuanced.

### Practical implications

The findings of this study provide key insights for sports marketers looking to leverage various types of endorsers for maximum marketing effectiveness. Traditional celebrity endorsements should remain a key strategy for high-visibility campaigns, particularly when the alignment between the endorser and the product is strong. SMIs may be more suitable than traditional celebrities when attempting to appeal to niche markets or when implementing campaigns involving niche products, where authenticity and relatability are crucial. Professional sport franchises should consider adopting endorsement strategies that emphasize traditional celebrities over SMIs. For example, traditional celebrities, such as popular singers and movie stars, could be featured at the opening ceremony or half-time performance of a professional sporting event. The findings of the present study indicate that brands must carefully consider the fit between product, endorser, and target audience to optimize the effectiveness of endorsement strategies.

### Limitations and future research

This study has several limitations. First, this research focuses on specific scenarios—a sports product and a spectator sporting event. The findings may not be generalizable to other scenarios. Future research should validate our findings by involving other product categories and sporting contexts. Second, the study did not perform random sampling; consequently, the sample is not highly representative. Future research may consider the issue of sample representativeness. Third, the lack of effective randomization in this research is a concern. The research is quasi-experimental. Fourth, using posters as experimental stimuli instead of videos somewhat diverges from the goal of exploring the essence of SMIs. The choice of posters stems from budget constraints that limit the feasibility of hiring SMIs for short video productions. Future studies should consider using videos instead of posters. Fifth, the sample size in Experiment 3 was smaller than that for the other experiments. Future studies should involve larger samples.

Overall, the present study uncovers the evolving dynamics of endorsement effectiveness in the digital age and reaffirms the enduring relevance of traditional celebrity endorsements. Future research should continue to explore the interplay between product–endorser fit and other moderating factors to fully understand the potential of both SMIs and traditional celebrities in sports marketing.

## Conclusion

The present study expands the literature by clarifying the distinct psychological mechanisms that drive the effectiveness of SMI and traditional celebrity endorsements in sports marketing, with conditioning and associative learning employed as core concepts. The findings indicate that traditional celebrities outperform SMIs in terms of endorsement effectiveness under conditions of high product–endorser fit, high popularity, low self-congruity, and low similarity. The contributions of this research are twofold. Theoretically, the findings enrich the literature on endorsement marketing. Practically, the findings provide valuable insights for stakeholders in participatory sports, spectator sports, and sporting goods in selecting endorsers who resonate most with the target audience. Further research could include other product categories and focus on a broader range of consumers to deepen our understanding of the evolving and increasingly influential role of digital influencers in sports marketing.

## Supporting information

S1 File Experiment 1. (XLSX)

S2 File Experiment 2. (XLSX)

S3 File Experiment 3. (XLSX)

S4 File Experiment 4. (XLSX)

S5 File Appendix 1 Factor Loadings of Measurement Items. (PDF)
