# Supplementary material for: Social media influencer or traditional celebrity? Moderational analysis on the relationship between endorser type and endorsement effectiveness
Source: PLoS One. 2025 Jul 1;20(7):e0326911. doi: 10.1371/journal.pone.0326911 (PMC12212533; doi:10.1371/journal.pone.0326911)
Supplement: S5 File — (PDF) [file pone.0326911.s005.pdf]

Appendix 1. Factor Loadings of Measurement Items.

	Experiment 1			Experiment 2			Experiment 3			Experiment 4		
	M	SD	FL	M	SD	FL	M	SD	FL	M	SD	FL
attitude(AD)	α = 0.922(0.959); AVE = 0.717(0.825)			α = 0.934(0.964); AVE = 0.747(0.844)			α = 0.973(0.989); AVE = 0.891(0.949)			α = 0.966(0.984); AVE = 0.851(0.930)		
AD 1	4.664 (4.205)	1.503 (1.854)	0.886 (0.952)	4.337 (3.885)	1.496 (1.851)	0.880 (0.939)	5.139 (4.545)	1.556 (1.931)	0.971 (0.983)	5.359 (4.961)	1.424 (1.776)	0.916 (0.969)
AD 2	4.893 (4.156)	1.297 (1.868)	0.845 (0.965)	4.705 (3.943)	1.418 (1.769)	0.860 (0.971)	5.129 (4.614)	1.488 (1.944)	0.942 (0.976)	5.477 (4.953)	1.441 (1.805)	0.907 (0.969)
AD 3	4.828 (4.459)	1.365 (1.637)	0.852 (0.896)	4.852 (4.303)	1.406 (1.621)	0.810 (0.910)	5.218 (4.703)	1.418 (1.847)	0.967 (0.974)	5.266 (5.031)	1.444 (1.774)	0.962 (0.978)
AD 4	4.049 (4.254)	1.700 (1.732)	0.791 (0.793)	3.549 (3.893)	1.846 (1.752)	0.890 (0.855)	4.990 (4.614)	1.526 (1.838)	0.868 (0.967)	5.000 (4.984)	1.521 (1.739)	0.884 (0.939)
AD 5	4.426 (4.180)	1.420 (1.676)	0.848 (0.918)	4.180 (3.910)	1.631 (1.585)	0.880 (0.913)	5.089 (4.653)	1.524 (1.900)	0.934 (0.968)	5.188 (4.883)	1.473 (1.764)	0.945 (0.956)
product-endorsers fit(F)	α = 0.973; AVE = 0.898			α = 0.982; AVE = 0.932			α = 0.993; AVE = 0.970			α = 0.990; AVE = 0.956		
F1	3.664	2.180	0.948	3.426	2.128	0.962	4.020	2.328	0.984	4.977	2.025	0.974
F2	3.795	2.069	0.972	3.451	2.073	0.989	4.069	2.246	0.981	4.867	2.060	0.991
F3	3.844	2.186	0.922	3.533	2.190	0.951	4.079	2.296	0.990	4.867	2.075	0.973
F4	3.754	2.118	0.952	3.451	2.081	0.962	4.000	2.293	0.991	4.922	2.076	0.984
popularity(P)	α = 0.918; AVE = 0.728			α = 0.898; AVE = 0.700			α = 0.947; AVE = 0.899			α = 0.972; AVE = 0.898		
P1	5.926	1.386	0.933	4.475	1.855	0.868	5.178	1.962	0.983	4.883	2.125	0.938
P2	5.852	1.406	0.964	4.566	1.641	0.982	5.168	1.939	0.987	4.984	1.952	0.973
P3	5.033	1.641	0.736	4.205	1.656	0.687	4.921	1.809	0.906	4.930	1.937	0.946
P4	5.008	1.491	0.756	4.262	1.536	0.776	5.000	1.811	0.907	4.898	1.898	0.934
self-congruity(SC)	α = 0.951; AVE = 0.770			α = 0.951; AVE = 0.770			α = 0.987; AVE = 0.929			α = 0.985; AVE = 0.922		
SC1	3.893	1.536	0.806	3.484	1.565	0.804	4.119	2.085	0.948	4.031	2.166	0.936
SC2	3.402	1.625	0.929	2.861	1.490	0.907	3.861	2.040	0.963	3.563	2.053	0.948
SC3	3.590	1.520	0.963	3.034	1.471	0.910	3.970	1.947	0.986	3.641	2.099	0.964
SC4	3.664	1.535	0.908	3.238	1.559	0.894	3.970	1.997	0.985	3.820	2.120	0.975
SC5	3.582	1.719	0.783	3.115	1.602	0.837	3.941	1.869	0.932	3.781	2.166	0.957

SC6	3.639	1.621	0.856	3.180	1.554	0.909	3.921	1.955	0.965	3.852	2.130	0.969
Similarity(S)	α = 0.972;<br>AVE = 0.961			α = 0.974;<br>AVE = 0.928			α = 0.990;<br>AVE = 0.967			α = 0.992;<br>AVE = 0.9801		
S1	3.270	1.631	0.963	2.779	1.480	0.947	3.881	1.925	0.985	3.516	2.027	0.993
S2	3.262	1.655	0.980	2.705	1.407	0.973	3.792	1.899	0.979	3.531	2.100	0.986
S3	3.287	1.624	0.935	2.738	1.401	0.968	3.792	1.915	0.990	3.461	2.088	0.989
Likeability(L)	α = 0.910;<br>AVE = 0.719			α = 0.949;<br>AVE = 0.821			α = 0.987;<br>AVE = 0.951			α = 0.967;<br>AVE = 0.880		
L1	5.279	1.268	0.797	4.943	1.386	0.854	5.287	1.687	0.960	5.336	1.589	0.904
L2	5.393	1.358	0.843	4.730	1.494	0.892	5.297	1.724	0.970	5.422	1.682	0.974
L3	5.459	1.306	0.876	4.885	1.495	0.952	5.277	1.778	0.990	5.477	1.537	0.951
L4	5.680	1.281	0.867	5.148	1.453	0.930	5.396	1.644	0.981	5.602	1.476	0.931

Note: Nonparenthesized values are pretest; parenthesized values are posttest; *M* = mean; *SD* = standard deviation; *FL* = factor loading; α = Cronbach's alpha; *AVE* = average variance extracted.
